# Therapeutic Effects of Electroacupuncture on Partial‐Thickness Supraspinatus Tear: Clinical Case Report

**DOI:** 10.1002/ccr3.73685

**Published:** 2026-10-09

**Authors:** Gholamreza Hajvalie, Ismail Ebrahimi Takamjani, Javad Sarrafzadeh, Hossein Saremi, Mohammad Reza Asadi

**Affiliations:** ^1^ Rehabilitation Research Center, Department of Physiotherapy, School of Rehabilitation Sciences Iran University of Medical Sciences Tehran Iran; ^2^ Department of Orthopedics, School of Medicine Hamadan University of Medical Sciences Hamadan Iran; ^3^ Department of Physiotherapy, School of Rehabilitation Sciences Hamadan University of Medical Sciences Hamadan Iran

**Keywords:** acupuncture, electroacupuncture, physiotherapy, rotator cuff, tendon injuries

## Abstract

This case report describes clinical and imaging improvement in a patient with a partial‐thickness supraspinatus tear following electroacupuncture combined with home exercise. These findings are hypothesis‐generating only and should not be generalized. Randomized controlled trials are required to determine efficacy, safety, and potential clinical applicability.

## 1 Introduction

Rotator cuff tears represent one of the most common musculoskeletal disorders affecting the shoulder, with the supraspinatus tendon being the most frequently involved structure [1]. The prevalence of rotator cuff tears increases with age, affecting approximately 20% of individuals in their 60's and more than 50% of those in their 80's [2]. While complete tears often require surgical intervention, partial‐thickness tears of the supraspinatus tendon present a therapeutic challenge, with no consensus on optimal management strategies [3].

Conservative treatment approaches for partial‐thickness supraspinatus tears typically include rest, physical therapy, non‐steroidal anti‐inflammatory drugs (NSAIDs), and corticosteroid injections [4, 5]. However, these conventional treatments often yield unsatisfactory outcomes, with studies reporting persistent pain and functional limitations in up to 40% of patients [6]. Additionally, corticosteroid injections, while providing short‐term pain relief, have been associated with potential detrimental effects on tendon healing and integrity [7].

In recent years, there has been growing interest in complementary and alternative medicine approaches for musculoskeletal disorders [8]. Electroacupuncture, a modified form of traditional acupuncture that incorporates electrical stimulation, has demonstrated promising results in treating various pain conditions and promoting tissue repair [9, 10]. The proposed mechanisms of EA include modulation of inflammatory mediators, enhancement of local blood flow, stimulation of fibroblast proliferation, and regulation of matrix metalloproteinase activity—all of which may contribute to improved tendon healing [11, 12].

Despite the theoretical basis for EA's potential efficacy in tendon healing, there is limited clinical evidence specifically addressing its application in partial‐thickness supraspinatus tears. Several randomized controlled trials have evaluated acupuncture for shoulder pain of various etiologies, but few have focused exclusively on specific rotator cuff pathologies or incorporated modern imaging techniques to objectively assess structural changes following treatment [13, 14].

This case report documents the clinical course and outcomes of a patient with a partial‐thickness supraspinatus tendon tear treated with a structured EA protocol. By integrating subjective outcome measures, functional assessments, and magnetic resonance imaging (MRI) findings, this report aims to provide insights into observed changes temporally associated with EA for this common shoulder condition and to inform the design of future controlled studies.

## 2 Case Presentation/Examination

This case report focuses on a 47‐year‐old right‐hand dominant female nurse patient who presented with a 24‐month history of left shoulder pain. The affected left shoulder was therefore her non‐dominant shoulder. Her occupational demands involved repetitive overhead reaching, lifting, and patient transfer activities with both arms; she reports the left (non‐dominant) side bore greater load when transferring patients which may have contributed to the etiology and persistence of her symptoms. The patient was selected based on the confirmed diagnosis of a partial‐thickness supraspinatus tendon tear (partial tear < 1 cm) via magnetic resonance imaging (MRI), previous failure of conventional conservative treatments (rest, 12 weeks of structured physiotherapy including ultrasound therapy, manual therapy, and a standardized rotator cuff strengthening program, 6 weeks of oral NSAIDs (diclofenac 50 mg) twice daily and two subacromial corticosteroid injections (methylprednisolone 40 mg) administered at 2‐month intervals), and absence of contraindications for electroacupuncture (e.g., bleeding disorders). This patient was not part of a prospective registry or a formal case series. Rather, she was selected retrospectively from routine clinical practice because she fulfilled predefined clinical criteria relevant to the purpose of this report, including MRI‐confirmed partial‐thickness supraspinatus tendon tear, failure of previous conservative management, complete adherence to the multimodal treatment program, and availability of comprehensive clinical and imaging follow‐up. In addition, the case was considered informative because both the clinical outcomes and serial MRI findings were well documented, allowing presentation of both symptomatic and structural changes over time. Other shoulder structures were unremarkable on the baseline MRI, showing no significant subacromial‐subdeltoid bursal thickening, a normal long head of the biceps tendon within the bicipital groove, and an intact acromioclavicular joint without joint space narrowing or significant osteophyte formation. In accordance with the institutional policy, formal ethics committee review is not required for retrospective case reports involving a single patient. Written informed consent was obtained from the patient for both treatment and publication of this case report, including accompanying images.

## 3 Methods

As part of routine clinical care, the patient underwent comprehensive baseline assessment, including history taking, physical examination, range of motion (ROM) measurement, pain intensity, and shoulder function evaluation. Range of motion was evaluated using a goniometer according to standardized protocols [15]. Shoulder function was assessed using Disabilities of the Arm, Shoulder and Hand (DASH) Questionnaire [16]. Pain intensity was measured using the numeric rating scale (NRS 0–10) [17]. The initial MRI confirmed a partial‐thickness supraspinatus tendon rupture (9 mm) without full‐thickness involvement. A timeline of the clinical course and key outcomes is presented in Table 1.

**TABLE 1:** Timeline of patient care and clinical outcomes.

Timeframe	Clinical event and interventions	Key outcome measures
Initial evaluation	baseline MRI confirming partial supraspinatus tear	NRS: 8/10, DASH: 72.5, Active Abduction: 95°
Weeks 1–6	Electroacupuncture (12 sessions, 2/week) + Daily Home Exercise Program	Progressed exercise resistance; good adherence (> 90%)
Week 6	Immediate post‐treatment evaluation	NRS: 3/10, DASH: 28.3, Active Abduction: 160°
Week 12	Occupational review	Gradual clearance and return to full work duties
6 Months	Final follow‐up evaluation and repeat MRI	NRS: 0/10, DASH: 12.3, Active Abduction: 175°; MRI: reduced tear size (~4 mm)

Electroacupuncture was performed by an expert physiotherapist in acupuncture using the electroacupuncture device (electronic acupuncture treatment instrument SDZ‐III, made in China). After sterilizing the skin, single‐use stainless steel acupuncture needles (0.25 mm × 30 mm, HUANQIU) were inserted into the following shoulder points: Large Intestine 16 (LI‐16), Triple Burner 14 (TB‐14), Small Intestine 10 (SI‐10), and an Ashi point at the tip of the coracoid process [18]. Point selection was based on traditional meridian theory and previously published protocols for shoulder disorders [18]. LI‐16 and TB‐14 are local points within the shoulder region believed to influence Qi flow in the affected area, while SI‐10 serves as an adjacent point. The Ashi point at the coracoid tip corresponds to the anatomical site of supraspinatus tendon insertion and was selected for focal stimulation. The electrodes were connected in such a way that the points LI‐16 and TB‐14 were connected together, and the point SI‐10 was connected to the front of the shoulder, the tip of the coracoid process [18]. After connecting the electroacupuncture device cables, a symmetric biphasic pulsed current at a fixed frequency of 5 Hz and pulse width (automatically defined by the SDZ‐III device) was applied for 20 min. The current intensity (up to 5 mA) was titrated individually to produce a strong but comfortable non‐painful paresthesia (De‐Qi sensation) without inducing forceful muscle contractions. To ensure safety, particularly near the thorax, needle insertion angle and depth at points LI‐16 and TB‐14 were carefully controlled to avoid pleural puncture, and needles were not oriented transversely towards the rib cage [18]. In total, 12 sessions of electroacupuncture treatment were performed (two sessions per week).

In addition to EA, the patient received instruction in a home exercise program focused on gentle range of motion and progressive rotator cuff strengthening exercises, performed daily as tolerated [6]. The program included:

- Week 1–2 (Phase 1—range of motion): Pendulum exercises (Codman's exercises), 3 sets of 10 repetitions, performed twice daily; passive shoulder flexion and external rotation using a cane or pulley, 3 sets of 10 repetitions, once daily.
- Week 3–6 (Phase 2—progressive strengthening): scapular retraction exercises; isometric shoulder abduction, flexion, and external rotation (3 sets of 10 repetitions, holding for 5 s, once daily); theraband external rotation and internal rotation (yellow resistance band, 3 sets of 15 repetitions, once daily). Exercises were progressed by increasing repetitions and resistance based on patient tolerance.
- Week 7–24 (Phase 3—maintenance): The patient was instructed to continue the strengthening exercises (3–4 times per week) and to maintain range of motion exercises as needed for symptom management.

Patient self‐reported adherence was documented in a weekly exercise log. The patient reported completing > 90% of prescribed sessions during Weeks 1–6 and continued the maintenance program 3–4 times per week between Week 6 and the 6‐month follow‐up. She reported modifying her work duties during the treatment period by avoiding overhead reaching and requesting assistance with patient transfers. These modifications were gradually reduced as her symptoms improved, and she had returned to full duties by the 3‐month follow‐up. No oral analgesics, anti‐inflammatory medications, or corticosteroid injections were administered during the treatment period.

## 4 Conclusion and Results

Outcomes measures (include pain intensity, Shoulder ROM, Shoulder function using DASH) were assessed at baseline, post‐intervention (Week 6), and 6‐month follow‐up (Table 2). Follow‐up MRI was performed at 6‐month using identical imaging parameters and sequences as the initial study. MRI images were reviewed by an orthopedic surgeon with a shoulder fellowship.

**TABLE 2:** Outcome measures.

Outcome	Baseline	Post‐intervention (Week 6)	6‐month follow‐up
Pain (NRS, 0–10)	8/10	3/10	0/10
DASH Score (0–100)	72.5	28.3	12.3
Active Abduction (°)	95	160	175
Active Flexion (°)	100	170	180
External Rotation (°)	25	50	60

Following the 6‐week electroacupuncture intervention, the patient demonstrated marked improvements in pain, function, and shoulder mobility. The patient demonstrated progressive reduction in pain throughout the treatment period. NRS scores decreased from 8/10 at baseline to 3/10 at Week 6, and 0/10 at the 6‐month follow‐up. This represented a 100% reduction in pain from baseline to final follow‐up. DASH score improved from 72.5 at baseline to 28.3 at Week 6, and 12.3 at 6‐month follow‐up, representing an overall 83% improvement. The observed improvement in DASH score from 72.5 to 12.3 exceeds the established minimal clinically important difference (MCID) of 10.8 points for shoulder disorders reported by Gummesson et al. [19] and Angst et al. [20], indicating that the functional improvement reported by this patient was clinically meaningful. Shoulder range of motion improved across all planes. Comparison of pre‐treatment and post‐treatment MRI scans revealed notable changes in the supraspinatus tendon (Figures 1 and 2). The follow‐up MRI at 6 months, using identical imaging parameters (oblique coronal T2‐weighted fat‐suppressed fast spin‐echo; 1.5T Siemens Magnetom Avanto; TR/TE = 3500/68 ms; slice thickness 3.5 mm), showed an estimated reduction in the dimensions from 9 mm (baseline) to approximately 4 mm, measured in the oblique coronal plane. The tear is specified as articular‐sided, partial‐thickness, involving < 50% of tendon thickness. A reduction in T2 signal hyperintensity within the tendon was also noted, which may be consistent with decreased tendinosis, though signal intensity was assessed qualitatively and was not normalized to a reference tissue. These imaging findings should be interpreted cautiously, as measurement of partial‐thickness tears is subject to variability depending on slice selection, sequence parameters, and reader interpretation. The reviewing surgeon was not blinded to the time point, representing a further limitation. No progression of the tear or new pathology was identified.

**FIGURE 1:**
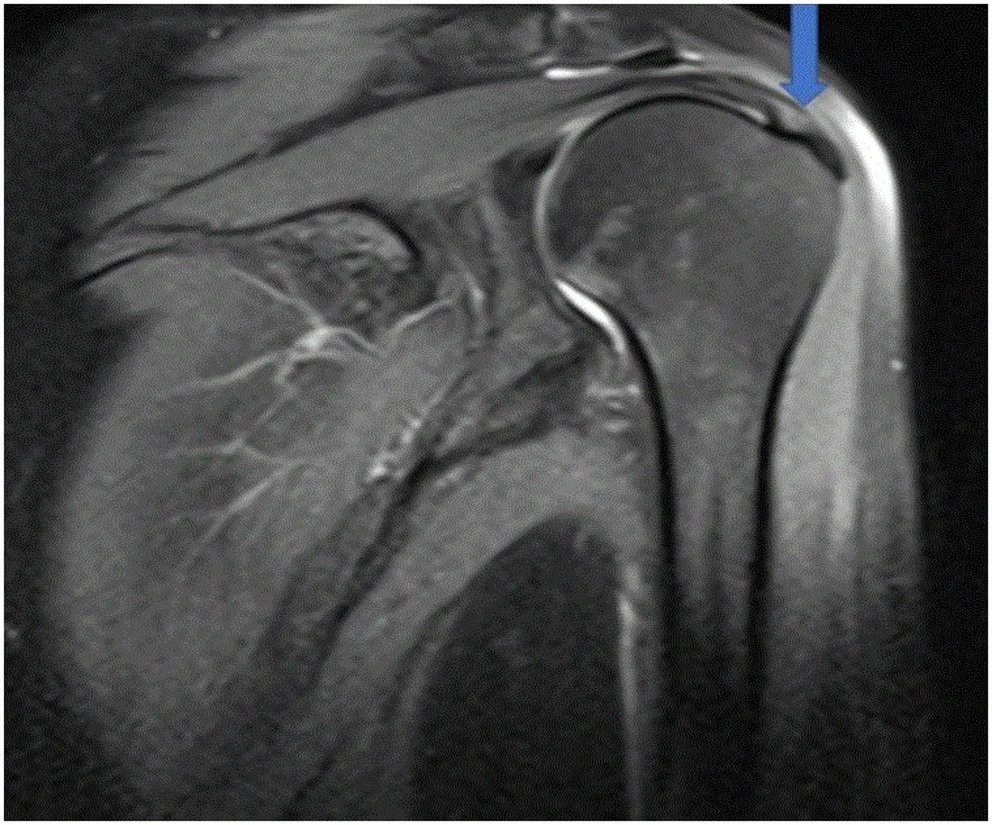
Oblique coronal T2‐weighted fat‐suppressed fast spin‐echo MRI (1.5 T, TR/TE = 3500/68 ms, slice thickness 3.5 mm) at baseline, showing increased signal intensity (arrow) in the articular‐sided supraspinatus tendon consistent with partial‐thickness tear and tendinosis.

**FIGURE 2:**
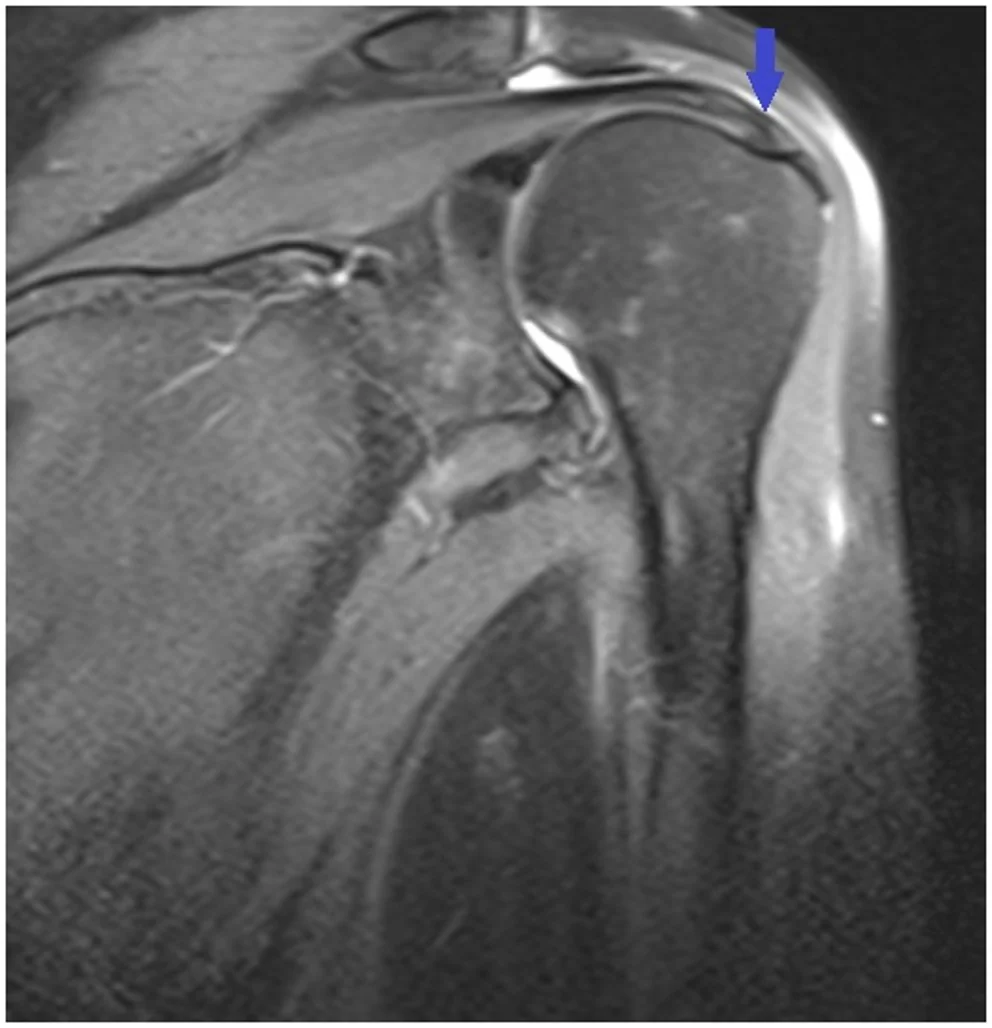
Oblique coronal T2‐weighted fat‐suppressed fast spin‐echo MRI (1.5 T, TR/TE = 3500/68 ms, slice thickness 3.5 mm) at 6‐month follow‐up, showing reduced signal intensity (arrow) at the same anatomic level, consistent with qualitative improvement in tendon appearance. This observation is descriptive only; signal intensity was not quantitatively normalized.

No adverse effects (e.g., infection, hematoma, or increased pain) were reported during or after EA sessions. Mild, transient soreness at needle insertion sites was reported after the first two treatment sessions, resolving spontaneously within 24 h. The patient reported that her primary goals were to return to pain‐free work and to avoid surgery. She described the treatment as “tolerable” and noted that she was able to resume normal activities by 3 months. She expressed satisfaction with the outcome and stated she would recommend the treatment to others with similar conditions.

## 5 Discussion

This case report describes observed clinical improvement and reduction in tear size noted on follow‐up MRI in one patient with a partial‐thickness supraspinatus tendon tear following a multimodal intervention that included electroacupuncture and a home exercise program. The patient experienced substantial pain reduction, functional recovery, and changes in tissue appearance on follow‐up MRI. These findings warrant discussion in the context of current understanding of rotator cuff pathology management and potential mechanisms of EA.

The clinical outcomes observed in this case compare favorably with those reported for conventional conservative treatments for partial‐thickness rotator cuff tears. Kukkonen et al. [5] reported satisfactory outcomes in approximately 75% of patients undergoing non‐operative treatment. However, the time to improvement typically ranges from 3 to 6 months, and residual symptoms are common [21]. Also, a systematic review by Giovannetti de Sanctis et al. [22] found that corticosteroid injections provide short‐term benefit for rotator cuff disease but with diminishing effects beyond 8 weeks and potential negative impacts on tendon integrity. In the present case, symptomatic and functional gains were maintained through the 6‐month follow‐up. However, this observation represents an individual clinical trajectory within a multimodal program (including home exercises) and cannot be interpreted as evidence of treatment superiority over other conservative strategies.

Our findings align with results from previous studies of acupuncture for shoulder pain, though few have specifically targeted confirmed rotator cuff tear. Johansson et al. [23] found that acupuncture combined with exercise produced greater functional improvement than ultrasound therapy, and Zhang et al. [24], in a meta‐analysis of randomized controlled trials, reported better clinical outcomes when acupuncture was added to exercise‐based physical therapy compared with physical therapy alone.

The partial reduction in tear size noted on follow‐up imaging is particularly noteworthy. The reduction in tear size in our patient is consistent with a healing response, though this cannot be confirmed, that merits consideration of potential mechanisms.

Several studies have investigated the effects of EA on tendon healing at the cellular and molecular levels. In a rat model of Achilles tendon rupture, Inoue et al. [11] demonstrated that EA increased fibroblast proliferation, collagen synthesis, and mechanical strength of healing tendons compared to controls. More recently, Song et al. [9] reported that EA reduced inflammatory cytokines (IL‐1β and TNF‐α) and increased anti‐inflammatory mediators (IL‐10) in injured rotator cuff tissues, potentially creating a more favorable environment for healing. De Almeida et al. [25] demonstrated that EA stimulation modulates matrix metalloproteinase (MMP) activity in tendon tissue, potentially enhancing extracellular matrix remodeling during the healing process.

The electrical component of EA may provide advantages over traditional acupuncture for tendon healing. Electrical currents have been shown to promote cellular migration, enhance blood flow, and stimulate growth factor production in connective tissues [26]. The frequency of 5 Hz utilized in our protocol aligns with research suggesting low‐frequency EA optimally stimulates release of endorphins and growth factors that may facilitate tissue repair [27].

As a single case report, this study has inherent limitations. Without a control comparison, we cannot definitively attribute the observed improvements to EA intervention alone. Natural history, placebo effects, and regression to the mean must be considered as potential contributing factors. Additionally, the concurrent implementation of a home exercise program introduces a co‐intervention that precludes isolation of EA‐specific effects.

The specific EA protocol utilized in this case, including point selection, stimulation parameters, and treatment frequency, was based on available evidence and clinical expertise but has not been systematically optimized. Different protocols might yield varying results, highlighting the need for further investigation of dose–response relationships in EA for tendon healing.

The most significant confounder in this report is the concurrent home exercise program. As EA and exercise were administered as a multimodal intervention, it is impossible to isolate the specific therapeutic effect attributable to EA alone. The observed improvements may reflect synergistic effects or may have been primarily driven by the rehabilitative exercises, with EA providing adjunctive pain relief that facilitated exercise adherence.

This case suggests that EA may be a valuable non‐invasive intervention for partial‐thickness supraspinatus tendon tears, particularly for patients who have not responded to conventional conservative measures or who wish to avoid corticosteroid injections or surgery. The minimal adverse events observed support the safety profile of EA when administered by qualified practitioners.

Future research should include randomized controlled trials comparing EA to standard conservative care, sham acupuncture, and other interventions for partial‐thickness rotator cuff tears. Such studies should incorporate both clinical and imaging outcome measures to evaluate symptom resolution and structural healing. Investigation of optimal EA parameters (frequency, intensity, and duration) and acupuncture point combinations would further refine treatment protocols.

Mechanistic studies exploring EA's effects on tendon cell biology, inflammation, and tissue remodeling would enhance understanding of observed clinical benefits. Correlation between biomarkers of tissue healing and clinical outcomes could potentially identify responder characteristics and optimize patient selection.

This case report describes observed clinical improvement and reduction in tear size noted on follow‐up MRI in one patient with a partial‐thickness supraspinatus tendon tear following a multimodal intervention that included electroacupuncture and a home exercise program. The patient experienced substantial reductions in pain and improvements in shoulder function and range of motion, with imaging changes consistent with possible tissue response. These outcomes suggest that a multimodal intervention including EA may be temporally associated with both symptomatic relief and tissue changes in rotator cuff pathology. However, due to the uncontrolled design and concurrent exercise, no causal conclusions can be drawn regarding EA‐specific efficacy. The findings support further investigation of EA as a non‐invasive intervention for partial rotator cuff tears and provide a foundation for future controlled studies to evaluate efficacy, optimal protocols, and physiological effects.

## Author Contributions

**Gholamreza Hajvalie:** conceptualization, data curation, methodology, writing – original draft. **Ismail Ebrahimi Takamjani:** supervision, writing – review and editing. **Javad Sarrafzadeh:** supervision, writing – review and editing. **Hossein Saremi:** data curation, methodology. **Mohammad Reza Asadi:** data curation, methodology, writing – original draft.

## Funding

The authors have nothing to report.

## Ethics Statement

Ethical approval from the Institutional Review Board was not necessary and was exempted for our investigation.

## Consent

The authors have obtained written informed consent from the patient. Informed consent was obtained from the patient for the publication of this case report and accompanying information.

## Conflicts of Interest

The authors declare no conflicts of interest.

## Data Availability

The data that support the findings of this study are available from the corresponding author upon reasonable request.

## References

[1] A. Yamamoto , K. Takagishi , T. Osawa , et al., “Prevalence and Risk Factors of a Rotator Cuff Tear in the General Population,” Journal of Shoulder and Elbow Surgery 19, no. 1 (2010): 116–120. 10.1016/j.jse.2009.04.006 19540777

[2] T. Teunis , B. Lubberts , B. T. Reilly , and D. Ring , “A Systematic Review and Pooled Analysis of the Prevalence of Rotator Cuff Disease With Increasing Age,” Journal of Shoulder and Elbow Surgery 23, no. 12 (2014): 1913–1921. 10.1016/j.jse.2014.08.001 25441568

[3] F. O. Lambers Heerspink , J. J. van Raay , R. C. Koorevaar , et al., “Comparing Surgical Repair With Conservative Treatment for Degenerative Rotator Cuff Tears: A Randomized Controlled Trial,” Journal of Shoulder and Elbow Surgery 24, no. 8 (2015): 1274–1281. 10.1016/j.jse.2015.05.040 26189808

[4] J. H. M. de‐Queiroz , M. B. de‐Medeiros , R. N. de‐Lima , and D. Q. Cerdeira , “Exercise for Rotator Cuff Tendinopathy,” Revista Brasileira de Medicina Do Trabalho: Publicação Oficial da Associação Nacional de Medicina Do Trabalho‐ANAMT 20, no. 3 (2022): 498–504. 10.47626/1679-4435-2022-698 PMC9904825 36793454

[5] J. Kukkonen , A. Ryösä , A. Joukainen , et al., “Operative Versus Conservative Treatment of Small, Nontraumatic Supraspinatus Tears in Patients Older Than 55 Years: Over 5‐Year Follow‐Up of a Randomized Controlled Trial,” Journal of Shoulder and Elbow Surgery 30, no. 11 (2021): 2455–2464. 10.1016/j.jse.2021.03.133 33774172

[6] J. E. Kuhn , “Exercise in the Treatment of Rotator Cuff Impingement: A Systematic Review and a Synthesized Evidence‐Based Rehabilitation Protocol,” Journal of Shoulder and Elbow Surgery 18, no. 1 (2009): 138–160. 10.1016/j.jse.2008.06.004 18835532

[7] M. T. Lin , C. F. Chiang , C. H. Wu , Y. T. Huang , Y. K. Tu , and T. G. Wang , “Comparative Effectiveness of Injection Therapies in Rotator Cuff Tendinopathy: A Systematic Review, Pairwise and Network Meta‐Analysis of Randomized Controlled Trials,” Archives of Physical Medicine and Rehabilitation 100, no. 2 (2019): 336–349. 10.1016/j.apmr.2018.06.028 30076801

[8] A. J. Vickers , E. A. Vertosick , G. Lewith , et al., “Acupuncture for Chronic Pain: Update of an Individual Patient Data Meta‐Analysis,” Journal of Pain 19, no. 5 (2018): 455–474. 10.1016/j.jpain.2017.11.005 PMC5927830 29198932

[9] W. Song , X. Han , K. Li , C. Chen , H. Wang , and X. Zheng , “Electro‐Acupuncture Promotes Repair of Rotator Cuff Injury in Rats,” Nan Fang Yi Ke Da Xue Xue Bao = Journal of Southern Medical University 40, no. 10 (2020): 1513–1517. 10.12122/j.issn.1673-4254.2020.10.19 PMC7606228 33118514

[10] Z. Y. Wang , Y. Zhang , H. Pei , C. Wang , and B. J. He , “Electroacupuncture Combined With Rehabilitation Techniques for Patients With Rotator Cuff Injury,” World Journal of Clinical Cases 13, no. 17 (2025): 98692. 10.12998/wjcc.v13.i17.98692 PMC11866268 40524768

[11] M. Inoue , M. Nakajima , Y. Oi , T. Hojo , M. Itoi , and H. Kitakoji , “The Effect of Electroacupuncture on Tendon Repair in a Rat Achilles Tendon Rupture Model,” Acupuncture in Medicine: Journal of the British Medical Acupuncture Society 33, no. 1 (2015): 58–64. 10.1136/acupmed-2014-010611 25335789

[12] R. Zhang , L. Lao , K. Ren , and B. M. Berman , “Mechanisms of Acupuncture‐Electroacupuncture on Persistent Pain,” Anesthesiology 120, no. 2 (2014): 482–503. 10.1097/ALN.0000000000000101 PMC3947586 24322588

[13] T. M. Allam , W. H. ElSayed , A. A. Azzam , and A. A. Nassif , “Effect of Electro‐Acupuncture in Chronic Rotator Cuff Tendonitis Patients: A Randomized Controlled Trial,” Egyptian Journal of Physical Therapy and Rehabilitation 4, no. 1 (2025): 29–38.

[14] Z. Y. Chen , M. H. Wang , and Z. Ye , “Effect of Electroacupuncture Combined With Rehabilitation Techniques on Shoulder Function in Patients With Rotator Cuff Injuries,” World Journal of Clinical Cases 12, no. 21 (2024): 4582–4589. 10.12998/wjcc.v12.i21.4582 PMC11235512 39070812

[15] C. C. Norkin and D. J. White , Measurement of Joint Motion: A Guide to Goniometry, 5e ed. (McGraw‐Hill Education, 2016).

[16] P. L. Hudak , P. C. Amadio , and C. Bombardier , “Development of an Upper Extremity Outcome Measure: The DASH (Disabilities of the Arm, Shoulder and Hand) [Corrected]. The Upper Extremity Collaborative Group (UECG),” American Journal of Industrial Medicine 29, no. 6 (1996): 602–608. 10.1002/(SICI)1097-0274(199606)29:6<602::AID-AJIM4>3.0.CO;2-L 8773720

[17] E. C. Huskisson , “Measurement of Pain,” Lancet 2, no. 7889 (1974): 1127–1131. 10.1016/s0140-6736(74)90884-8 4139420

[18] G. Gyer , J. Michael , and B. Tolson , Dry Needling for Manual Therapists: Points, Techniques and Treatments, Including Electroacupuncture and Advanced Tendon Techniques (Jessica Kingsley Publishers, 2016).

[19] C. Gummesson , I. Atroshi , and C. Ekdahl , “The Disabilities of the Arm, Shoulder and Hand (DASH) Outcome Questionnaire: Longitudinal Construct Validity and Measuring Self‐Rated Health Change After Surgery,” BMC Musculoskeletal Disorders 16, no. 4 (2003): 11, 10.1186/1471-2474-4-11. PMC165599 12809562

[20] F. Angst , H. K. Schwyzer , A. Aeschlimann , B. R. Simmen , and J. Goldhahn , “Measures of Adult Shoulder Function: Disabilities of the Arm, Shoulder and Hand Questionnaire (DASH) and Its Short Version (QuickDASH), Constant‐Murley Score (CMS), and the Shoulder Pain and Disability Index (SPADI),” Arthritis Care & Research 63, no. 11 (2011): S174–S188. 10.1002/acr.20630 22588743

[21] S. Moosmayer , G. Lund , U. S. Seljom , et al., “Fifteen‐Year Results of a Comparative Analysis of Tendon Repair Versus Physiotherapy for Small‐To‐Medium‐Sized Rotator Cuff Tears: A Concise Follow‐Up of Previous Reports,” Journal of Bone and Joint Surgery. American Volume 106, no. 19 (2024): 1785–1796. 10.2106/JBJS.24.00065 PMC11594065 39197154

[22] E. Giovannetti de Sanctis , E. Franceschetti , F. De Dona , A. Palumbo , M. Paciotti , and F. Franceschi , “The Efficacy of Injections for Partial Rotator Cuff Tears: A Systematic Review,” Journal of Clinical Medicine 10, no. 1 (2020): 51. 10.3390/jcm10010051 PMC7795404 33375716

[23] K. M. Johansson , L. E. Adolfsson , and M. O. Foldevi , “Effects of Acupuncture Versus Ultrasound in Patients With Impingement Syndrome: Randomized Clinical Trial,” Physical Therapy 85, no. 6 (2005): 490–501. 15921471

[24] H. N. Zhang , J. G. Chen , X. Y. Wang , et al., “Efficacy Comparison Between Acupuncture and Other Modalities in the Treatment of Rotator Cuff Diseases: Meta‐Analysis of Randomized Controlled Trials,” American Journal of Translational Research 16, no. 2 (2024): 599–616. 10.62347/NCRJ1270 PMC10918137 38463603

[25] M. S. de Almeida , A. A. de Aro , F. D. R. Guerra , C. P. Vieira , B. de Campos Vidal , and E. Rosa Pimentel , “Electroacupuncture Increases the Concentration and Organization of Collagen in a Tendon Healing Model in Rats,” Connective Tissue Research 53, no. 6 (2012): 542–547. 10.3109/03008207.2012.710671 22891942

[26] Z. Q. Zhao , “Neural Mechanism Underlying Acupuncture Analgesia,” Progress in Neurobiology 85, no. 4 (2008): 355–375. 10.1016/j.pneurobio.2008.05.004 18582529

[27] J. S. Han , “Acupuncture and Endorphins,” Neuroscience Letters 361, no. 1–3 (2004): 258–261. 10.1016/j.neulet.2003.12.019 15135942

